# The Evaluation of P‐Wave Parameters in Patients With Percutaneous Closure of Atrial Septal Defect

**DOI:** 10.1111/anec.70076

**Published:** 2025-04-08

**Authors:** Ramazan Astan, Fehmi Kacmaz, Ersin Saricam, Erdogan Ilkay

**Affiliations:** ^1^ Batman Training and Research Hospital Batman Turkey; ^2^ Department of Cardiology, Faculty of Medicine Uskudar University İstanbul Turkey; ^3^ Ozel Nev Hospital Sanlıurfa Turkey; ^4^ Medicana International Ankara Hospital, Cardiology Clinic Atilim University Ankara Turkey; ^5^ Medicana International Ankara Hospital Cardiology Clinic Ankara Turkey

**Keywords:** atrial septal defect, electrocardiography, P‐wave dispersion, P‐wave duration

## Abstract

**Background:**

Atrial septal defect (ASD) can lead to volume overload and related changes in P‐wave parameters in surface electrocardiograms of these patients. In this study, we aimed to evaluate the effect of volume overload on P‐wave parameters in patients with ASD.

**Materials and Methods:**

This study is a retrospective cohort analysis. A total of 142 patients with secundum ASD who underwent percutaneous closure were evaluated. P‐wave duration (Pmax) and P‐wave dispersion (PWD) were measured on the surface ECG before and 1 h after the closure procedure. We evaluated P‐wave parameters in terms of defect size, duration of the volume overload, and closure device sizes.

**Results:**

Pmax and PWD were significantly decreased after the procedure compared with the values before the procedure (*p* < 0.001). Pmax values had a statistically significant correlation with ASD size (< 20 mm or ≥ 20 mm) both before and after the procedure. Pmax values were significantly higher in patients older than 30 years of age (119.6 ± 19.5 vs. 102.7 ± 17.1 ms, respectively; *p* = 0.039). A significantly positive correlation was found between pre‐ and post‐procedural Pmax and defect sizes (*r* = 0.474, *p* = 0.019 and *r* = 0.4233, *p* = 0.04, respectively). However, no positive correlation between PWD and defect age and size was present.

**Conclusion:**

Percutaneous closure of ASD is associated with an immediate decrease in both Pd and Pmax that seems to be related to the acute volume overload cessation in cardiac chambers.

## Graphical abstract

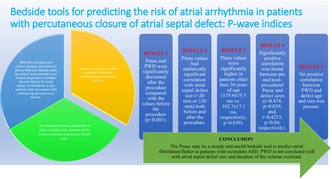

## 1 Introduction

Atrial septal defect (ASD) is one of the most common congenital heart defects (van der Linde et al. 2011). In clinical presentation, atrial arrhythmias have been frequently observed. The prevalence of atrial tachyarrhythmia in patients with unrepaired ASD is higher than that of the healthy population (Baumgartner et al. 2021; Muroke et al. 2023).

In patients with ASD, several risk factors and conditions have been identified to contribute to the development and progression of AF, including the size of the shunt, pulmonary hypertension, and age (Chubb et al. 2014). Arrhythmogenesis in patients with ASD appears to be multifactorial (Parameswaran et al. 2020). It is well known that left‐to‐right atrial shunting leads to right atrial (RA) and right ventricular (RV) because of chronic diastolic volume overload. Structural remodeling in the RA is time dependent, which has already been demonstrated in previous histological studies (Ueda et al. 2013). Furthermore, the age of the patient at the time of the ASD closure is one of the main predictors of persistent or new arrhythmias (Vyas et al. 2020).

P‐wave dispersion (PWD) is the difference between the maximum and the minimum P‐wave duration recorded from multiple ECG leads; increased P‐wave duration and PWD show prolongation of intra‐ and inter‐atrial conduction time and are associated with the heterogeneity in atrial propagation of sinus impulses (Geng et al. 2014). Importantly, these parameters are simple ECG markers that can also be used to identify patients with AF.

At present, secundum‐type ASD is usually treated with transcatheter device closure, and the long‐term effects of ASD closure on P‐wave duration have been well documented (O'Neill et al. 2020). However, information about the acute effects of ASD closure on P‐wave is limited.

In this study, we aimed to evaluate the risk factors of atrial flutter or fibrillation by using these simple ECG parameters in patients with secundum ASD undergoing percutaneous closure.

## 2 Methods

The present study is a retrospective cohort analysis. A total of 142 patients with secundum ASD who underwent percutaneous closure were evaluated. Informed consent was obtained from each subject, and the study was approved by the local ethics committee of Batman Public Hospital (no: 85017011‐123/11.01.2019).

Among the indications for secundum ASD closure are a pulmonary‐to‐systemic flow ratio of 1.5:1 or higher, echocardiographic evidence of right atrial and right ventricular enlargement, and reversible pulmonary hypertension. All patients underwent successful percutaneous ASD closure, and none of them had previously documented arrhythmias. Demographic data of the participants, including age and gender, were recorded. All study participants were in sinus rhythm during the evaluation. The patients were evaluated in terms of P‐wave duration, PWD measured from the pre‐ and post‐procedural ECG recordings, duration of volume overload, and defect size. Modified Simpson's method was used to determine left ventricular [LV] ejection fraction and LV dysfunction based on the current guidelines (Mitchell et al. 2019). Atrial septal defect sizes of the patients were measured by a single cardiologist via transesophageal echocardiography (TEE) GE Vingmed Vivid 5 echocardiography device (GE Vingmed Ultrasound, Horten, Norway).

The exclusion criteria were primum or sinus venosus ASD, antiarrhythmic medications and LV dysfunction. None of the patients had diseases affecting P‐wave durations, such as systemic hypertension, history of arrhythmia, chronic obstructive pulmonary disease, coronary heart disease, active infection or thyroid dysfunction.(20 patients).

### 2.1 Device Implantation

ASD closure procedures were performed under the guidance of TEE. In all subjects, either Occlutech Figulla or Amplatzer septal occluder devices were used to close the defect. All operations were performed under general anesthesia. During the process, the blood pressure and heart rate did not change significantly in any of the patients.

### 2.2 Electrocardiographic Measurements

The standard 12‐lead ECGs were obtained in a comfortable supine position by using a recorder (MAC 2000, GE Medical Systems, Milwaukee, WI, USA) with a paper speed of 50 mm/s and amplification of 1 mV/10 mm. ECG was recorded before the procedure and repeated 1 h after the procedure in each patient.

P‐wave was defined as the distance from the point of the first visible upward or downward departure from the baseline to the point of return to the baseline. The average P‐wave of three consecutive beats from each lead was determined. If the P‐wave was measurable in at least nine leads, the patient was kept for further analysis. The maximum P‐wave duration was defined as the longest P‐wave duration for all the derivations. PWD was defined as the difference between the maximum and minimum P‐wave durations for all 12 leads. P‐wave measurements were performed by two investigators who were unaware of the subject's data. Intra‐observer and inter‐observer coefficients of variation were 3.3% and 4.7%, respectively.

All of the same measurements were repeated 1 h after the closure procedure, thereby recording the longest P‐wave and PWD in the pre‐ and post‐procedure ECGs.

### 2.3 Statistical Analysis

All statistical analyses were performed using SPSS (Statistical Package for Social Sciences) 18.0 (SPSS, Chicago, IL, USA). All continuous variables are presented as mean ± SD (standard deviation). The Student's *t*‐test was used to assess the associations of qualitative variables between the two groups of patients. We used the Wilcoxon matched‐pairs test to analyze changes in the quantitative variables before and after the procedure. Regression analysis was performed to detect independent variables affecting PWD. Moreover, the Spearman correlation was used to detect any correlation between hemodynamic variables and P‐wave parameters. A *p* < 0.05 was considered statistically significant.

## 3 Results

The baseline clinical, laboratory, and echocardiographic characteristics of the 142 patients with secundum ASD are presented (Table 1). The mean age of the patients was 38.2 ± 19.5 years, and 73% of patients were female (104 patients). All patients had a normal left ventricular ejection fraction (63.4% ± 7.6%). Systolic pulmonary artery pressures and pulmonary artery diameter were increased (39.7 ± 14.4 mmHg and 26.1 ± 6.3 mm, respectively). Mean defect and closure device sizes were 21.8 ± 11.5 and 23.9 ± 13.8 mm, respectively.

**TABLE 1:** Baseline characteristics of study populations (*n* = 142).

Age, (years)		38.2 ± 19.5	
Female, *n* (%)		104 (73.2)	
Defect size, mm		21.8 ± 11.5	
Left ventricle ejection fraction, %		63.4 ± 7.6	
Left atrium diameter, mm		37.1 ± 11.3	
Right atrium diameter, mm		40.5 ± 13.2	
Systolic pulmonary artery pressure, mmHg		39.7 ± 14.4	
Pulmonary artery diameter, mm		26.1 ± 6.3	
Device diameter used, mm		23.9 ± 19.5	
**P wave parameters**	**Before**	**After**	***p***
Pmax, ms	113.3 ± 20	98.2 ± 13.5	< 0.001
Pmin, ms	53.9 ± 10.7	62.3 ± 11.9	< 0.001
PWD, ms	59.4 ± 19.4	38.3 ± 12.3	< 0.001

Abbreviations: mm, millimeter; ms, millisecond; Pmax, maximum P‐wave duration; Pmin, minimum P‐wave duration; PWD, P‐wave dispersion.

Before the procedure, Pmax was 113.3 ± 20.1 ms, Pmin was 53.9 ± 10.7 ms, and PWD was 59.4 ± 19.4 ms. After the procedure, Pmax was 98.2 ± 13.5 ms, Pmin was 62.3 ± 11.9 ms, and PWD was 38.3 ± 12.3 ms (*p* < 0.001).

We further divided the patients into two subgroups according to the ASD size (< 20 mm and ≥ 20 mm) and compared the respective ECG parameters. There was a statistically significant change in Pmax values before and after the procedures. Before the procedure, the Pmax values of the patients with defect sizes larger than 20 mm were 124.4 ± 18.7 ms, whereas the Pmax values of those with smaller defect sizes (< 20 mm) were 106.6 ± 18.2 ms (*p* = 0.037). However, PWD values did not change significantly before and after the procedure (65.5 ± 17.2 vs. 52.6 ± 18.8 ms, respectively; *p* = 0.106) (Table 2).

**TABLE 2:** Comparison of patients ECG parameters according to atrial septal defect size (Pmax, PWD).

	Defect size		*p*
	< 20 mm (*n* = 64)	> 20 mm (*n* = 78)	
Pre‐procedure			
Pmax, ms	106.6 ± 18.2	124.4 ± 18.7	0.037
PWD, ms	52.6 ± 18.8	65.5 ± 17.2	0.106
Post‐procedure			
Pmax, ms	94.6 ± 13.1	109.4 ± 11.8	0.011
PWD, ms	36.3 ± 9.1	41.6 ± 16.3	0.389

Abbreviations: mm, millimeter; ms, millisecond; Pmax, maximum P‐wave duration; Pmin, minimum P‐wave duration; PWD, P‐wave dispersion.

Analysis regarding the impact of the duration of volume overload on P wave indices revealed that Pmax values were significantly higher in patients older than 30 years of age compared with the younger ones (119.6 ± 19.5 vs. 102.7 ± 17.1 ms, respectively; *p* = 0.039) (Table 3).

**TABLE 3:** Comparison of patients based on volume overload duration (age).

	Age		*p*
	< 30 years (*n* = 28)	> 30 years (*n* = 114)	
Pre‐procedure			
Pmax, ms	102.7 ± 17.1	119.6 ± 19.5	0.039
PWD, ms	55.0 ± 17.5	59.0 ± 20.2	0.616
Post‐procedure			
Pmax, ms	92.7 ± 14.8	104.6 ± 12.6	0.063
PWD, ms	37.2 ± 13.0	39.0 ± 12.2	0.745

Abbreviations: mm, millimeter; ms, millisecond; Pmax, maximum P‐wave duration; Pmin, minimum P‐wave duration; PWD, P‐wave dispersion.

Spearman correlation analysis revealed a significant positive correlation between pre‐ and post‐procedural Pmax duration and defect size (*r* = 0.474, *p* = 0.019; and, *r* = 0.4233, *p* = 0.04; respectively) (Table 4). We found no positive correlation between PWD and device size.

**TABLE 4:** Correlation analyses between device closure size used and P wave parameters.

P wave measurements	*r*	*p*
Pre‐procedure		
Pmax	0.474	0.019
PWD	0.503	0.007
Post‐procedure		
Pmax	0.4233	0.040
PWD	0.360	0.084

Abbreviations: mm, millimeter; ms, millisecond; Pmax, maximum P‐wave duration; Pmin, minimum P‐wave duration; PWD, P‐wave dispersion; *r*, Spearman correlation coefficients.

## 4 Discussion

The volume overload on the right heart chambers in patients with ASD contributes to the prolongation of P‐wave duration. It has been shown that Pmax and PWD are significantly longer in adult patients with ASD (Guray et al. 2003; Pole Rio et al. 2019).

P wave abnormalities detected by ECG have been thought to reflect right atrial enlargement and altered conduction in the atrial tissue (Nitta et al. 2022). PWD ≥ 40 ms is associated with heterogeneous electrical activity in different regions of the atrium that might cause atrial tachyarrhythmias. The volume overload in the right heart chamber leads to geometrical and electrical distortions in cardiac tissues (Veldtman et al. 2001). The patients with ASD have relatively higher Pmax and PWD values due to the longstanding effect of volume overload. After closure, the sudden resolution of volume overload may be the leading cause of the immediate shortening of Pmax and a decrease in PWD (Santoro et al. 2004; Lelakowska et al. 2018). Veldtman et al. showed significant improvement in right heart morphology within 1 month, along with positive mechanical and electrophysiological changes, after device closure in patients with ASD (Veldtman et al. 2001).

Thilén et al. reported that P‐wave prolongation was related to delayed conduction rather than atrial size and concluded that prolonged Pmax and increased PWD could represent mechanical and electrical changes of atrial myocardium in patients with ASD and that those changes might be more prominent with greater shunt volumes in patients with larger septal defects (Thilén et al. 2007).

In the present study, we found a rapid positive change in the P‐wave parameters (P max and PWD). Pmax values were significantly higher in patients older than 30 years than in younger patients. The PWD also tended to be higher in older patients, but this association was not statistically significant. This finding indicated that Pmax may be related to the duration of volume overload. We observed that the mean Pmax and PWD decreased after device closure. These acute changes may be related to sudden overload resolution. Furthermore, we demonstrated a positive correlation between Pmax and the defect size. However, the same did not hold true for the defect size and PWD.

It has been known that ASD can cause volume overload in the right heart and this volume overload is likely to be responsible for the elongation of Pmax. Therefore, the treatment of ASD with percutaneous closure reduces volume overload, thereby shortening Pmax (Lelakowska et al. 2018).

Although AF can be completely asymptomatic, approximately two‐thirds of patients experience at least intermittent symptoms, which can be disabling or even markedly impair their health‐related quality of life. Specifically, stroke is the first manifestation of previously unknown AF in > 25% of AF‐related strokes (Yiin et al. 2017).

Patients with ASD have an increased risk of atrial fibrillation, stroke, and migraines. The rate of new‐onset atrial fibrillation remained elevated even after ASD closure (Muroke et al. 2023). P‐wave duration and PWD are non‐invasive ECG markers for atrial remodeling and predictors of AF (Uggen Rasmussen et al. 2020; Panagiota et al. 2023). Therefore, increased P duration time (Pmax) and PWD are powerful markers for predicting the development of atrial fibrillation. In this regard, Pmax could prove to be an important bedside tool for predicting atrial arrhythmia during the follow‐up of patients with ASD.

The main limitation of our study is that the P wave indices were not analyzed by a software program but were rather manually using a magnifying lens.

## 5 Conclusion

Percutaneous closure of ASD is associated with an immediate decrease in both Pd and Pmax that seems to be related to the acute volume overload cessation in cardiac chambers. Pmax is related to the defect size and duration of the volume overload. The improvement in electrocardiographic P wave indices just after percutaneous closure of ASD may reduce atrial arrhythmogenic burden. We need large‐scale studies to test this comprehensively.

## Author Contributions

Substantial contributions to the conception or design of the work; or the acquisition, analysis, or interpretation of data for the work: Ramazan Astan, Fehmi Kacmaz. Drafting the work or revising it critically for important intellectual content: Fehmi Kacmaz, Ersin Saricam, and Erdogan Ilkay. Final approval of the version to be published: Ramazan Astan, Fehmi Kacmaz, and Erdogan Ilkay. Agreement to be accountable for all aspects of the work in ensuring that questions related to the accuracy or integrity of any part of the work are appropriately investigated and resolved: Ramazan Astan, Fehmi Kacmaz, Ersin Saricam, and Erdogan Ilkay.

## Ethics Statement

Our study was approved by the local ethical committee of Batman Public Hospital (no: 85017011‐123/11.01.2019).

## Conflicts of Interest

The authors declare no conflicts of interest.

## Data Availability

All relevant data are within the paper and its Supporting Information files.
